# PROSPECTIVE ASSOCIATIONS BETWEEN THE STATE MINIMUM WAGE AND COGNITIVE FUNCTION IN OLDER AMERICANS

**DOI:** 10.1093/geroni/igae098.4371

**Published:** 2024-12-31

**Authors:** Boram Lee, Lucia Petito, Daniel Kim

**Affiliations:** Northeastern University, Boston, Massachusetts, United States; Northwestern University, Chicago, Illinois, United States; Northeastern University, Boston, Massachusetts, United States

## Abstract

Limited research has explored how social policies influence cognitive health in older age. This study aimed to examine the associations between the state-level minimum wage and cognitive function in older US adults. We used data from the Health and Retirement Study (1998-2020) on non-self-employed workers who were age 50-75 and free of dementia at baseline, with cognitive function measured during the study period (N=7490). For cognitive function, we used data based on the Telephone Interview for Cognitive Score (TICS; 0-27, with higher values indicating better cognitive function). We explored the state-level minimum wage, adjusted to 2021 US dollars, lagged by 2, 6, and 10 years. We employed individual fixed-effects linear models and examined for differences in associations by wage, working hours, race/ethnicity, and sex. Among all workers, the state minimum wage was not associated with cognitive function. However, for low-wage workers and part-time workers, a $1 increase in the 6-year lagged state minimum wage was associated with a 0.40 (95% CI=0.17 to 0.64, p=.001) and 0.17 (95% CI=0.03 to 0.32, p=.01) point increase in the TICS, respectively, controlling for time-invariant individual factors and time-varying individual-level and state-level covariates. Among low-wage workers, stronger associations between the state minimum wage and increases in cognitive function were observed in non-Hispanic Whites (β= 0.43, p<.001) but not Black Americans (β=0.04, p=0.91) and Hispanic Americans (β=0.30, p=0.21). There were no differences by sex. Overall, our findings support that increasing the state minimum wage could benefit the cognitive health of older adults, particularly among low-wage workers.

